# Explanation of the Rigor in Puerperal Women Immediately after Birth of Child

**Published:** 1875-03

**Authors:** 


Explanation of the Rigor in Puerperal Women Immediately after Birth of Child.—Pofanukuch (*Archives of Gynæcology, American Journal of Medical Sciences,*) October, 1874 gives his reasons for being dissatisfied with the ordinary explanations of the above peenomenon. These explanations are sudden loss of blood, sudden withdrawal of the blood from the surface, caused by the emptying of the uterus, and the consequent lessening of the pressure to which the great vessels have been subjected; the exposure of the woman during the latter parts of labor, or her lying in sheets damp with the escape of the water. Pofanukuch quotes the experiments of Wurster, showing that the temperature of the fœtus in utero is at least 0.9° higher than that of the mother. Thus the pregnant woman has a second centre of warmth in her uterus, but as her own temperature is not increased, she must be producing less warmth than if not pregnant. The moment the child is born this centre of warmth is removed, and there is a disproportion between the amount of heat produced and given off. The effort to bring about an equilibrium causes the rigor. The intestines are most susceptible to heat and cold, and the source of heat is taken from direct contact with them. The uterus also has so sunk down that the intestines come into contact with the thinned abdominal wall, and so suffer further loss of heat.—*Detroit Review of Medicine and Pharmacy.*

